# Pathways to integrated learning outcomes in professional and entrepreneurship education: Evidence from student perceptions

**DOI:** 10.1371/journal.pone.0358648

**Published:** 2026-09-21

**Authors:** Jian Zhang, Khalid Usman, Hongmei Yang, Qianqian Yan

**Affiliations:** 1 School of Entrepreneurship Management, Sanming University, Sanming, Fujian, China; 2 School of Economics and Management, Sanming University, Sanming, Fujian, China; Alexandru Ioan Cuza University: Universitatea Alexandru Ioan Cuza, ROMANIA

## Abstract

Drawing on the resource-based view, constructivist learning theory, and the theory of planned behavior, this study develops a process-oriented framework conceptualized as “resource input-cognitive construction-behavioral engagement” to examine how integrated learning outcomes in professional and entrepreneurship education are associated with multiple educational factors. Using survey data from 584 university students, the empirical analysis indicates that teaching content, teacher competence, student engagement, and university-enterprise resources are all positively associated with integrated learning outcomes. These associations are primarily reflected through instructional practice effectiveness as a key process variable. In contrast, policy support shows no significant direct association with learning outcomes but appears to be related through indirect pathways. By adopting a process perspective, this study highlights how educational resources, instructional practices, and student engagement jointly relate to learning outcomes in integrated education contexts. The findings offer implications for improving curriculum design, strengthening practice-oriented teaching, enhancing teacher competence, and promoting university enterprise collaboration in higher education.

## 1 Introduction

In the context of the dramatic changes in global economic structures and the rapid advances in science and technology, innovation is now regarded as an important manifestation of countries’ core competitiveness. The need for talented individuals who can innovate and engage in entrepreneurship has become increasingly strong [1]. However, traditional professional education primarily focuses on the systematic transmission of knowledge and the cultivation of professional skills, emphasizing the integrity of the disciplinary system and its connection to vocational abilities. There is a deficiency in cultivating students’ innovative spirit, entrepreneurial awareness, and cross-border integration ability [2]. Innovation and Entrepreneurship Education (IEE) has been an important direction of the education system reform in recent years. It is committed to stimulating students’ innovative consciousness, enhancing their entrepreneurial capabilities, and promoting the transformation of knowledge and the creation of social value [3]. Meanwhile, its educational goals emphasize the individual’s active exploration, critical thinking, and practical skills, which complement the subject-centered professional education. Therefore, promoting the deep integration of professional education and innovation and entrepreneurship education has become one of the key paths for higher education reform [4].

In response to the national “innovation-driven development” strategy and the policy orientation of “mass entrepreneurship and innovation”, Chinese universities have successively initiated integrated education reforms in recent years. Various models of “integration of professional education and innovation,” such as course embedding, practice-driven approaches, and school-enterprise collaboration, have been explored. Although these practices have achieved positive results to a certain extent, most remain at the level of case summaries and model demonstrations. It lacks systematic theoretical support and in-depth analysis at the mechanism level. In particular, how different educational elements interact within the integration process and how specific factors relate to integrated learning outcomes remain insufficiently theorized, with no widely applicable framework yet established [5].

While current institutional practices have yielded positive outcomes, they primarily remain at the level of case summaries and experiential model demonstrations, lacking systematic theoretical grounding and in-depth mechanism-level analysis [4,5].

Three crucial limitations are found within the current literature review. First, there is a distinct lack of quantitative measures of synergy of combining professional and entrepreneurship education. Second, more importantly, the interrelationships among different factors, especially the mechanisms of resource-to-outcome transformation, have never been empirically studied [2]. As a result, the field has become fragmented: it knows what is important (content, teachers, etc.), but does not understand how these elements work together to produce results for students. This practice-theory gap greatly hinders further improvements in this area of research.

To fill these gaps, this research presents a new theoretical framework named “Resource Input-Cognitive Construction-Behavioral Engagement,” which is empirically validated for the first time. Based on the RBV, CLT and TPB theories, this new theoretical framework argues that integration is not merely a simple accumulation of institutional resources but a dynamic process in which inputs of institutional resources are converted into learning outcomes through certain pedagogical approaches and students’ engagement behaviors. In other words, we conceptualize Instructional Practice Effectiveness as the important mediating variable, the “Cognitive Construction” engine that transforms tangible resources (content, teacher competence, enterprise collaboration, policy support) and students’ behavioral disposition (engagement) into Perceived Integration Outcomes.

By employing this perspective and an empirical investigation of the theorized mediating variables, our study contributes to theory by demonstrating the mechanism underlying the generation of integrated learning. It gives a better insight into the nature of the relationship between educational resources, teaching methods, and learner engagement in achieving success. In practice, it will provide recommendations for curriculum planning, faculty development, resource allocation, and policy implementation in higher education.

## 2 Theoretical foundations and research hypotheses

### 2.1 Theoretical foundations

A multi-theoretic approach would help understand the learning processes that result from an integrated professional and entrepreneurial education. However, the current literature shows that there is a tendency towards theoretical insularity-researchers usually utilize one particular theoretical approach for examining a certain aspect of the integration process: resource-based view (RBV), constructivist learning theory (CLT), or theory of planned behavior (TPB) [6–8]. Although each approach provides useful insight, their individual use has led to a fragmented understanding of how inputs become outputs. There are three major theoretical gaps.

First, RBV offers an excellent perspective for analyzing the institutional resources (such as teaching content, teachers’ competence, enterprise collaboration and policy support) required to achieve competitive educational advantages [6,9]. But its predominant approach is inherently static and input-focused. While it clarifies the existence of resources, it does not offer any insight into the dynamics of using, organizing and converting these resources into expected results [9]. From an educational perspective, it represents a significant drawback, as it fails to analyze the process of using existing resources to achieve the intended outcome. For example, RBV could reveal the presence of high-quality teaching content or competent teachers at the university, but it does not provide any understanding of how these resources are used in classrooms to produce learning outcomes. The limitation of the theory is that it can oversimplify educational reform into a “resource accumulation” approach and neglects the learning process, which is the core of education.

Secondly, CLT provides an effective response to this view by highlighting the learning process [7,10]. In essence, CLT is based on the epistemological assumption that knowledge is created through active engagement in practice and social interaction [11,12]. As such, CLT stresses the importance of creating opportunities for meaningful learning, where students engage in meaningful activities, problem-solving, and receive feedback [13]. However, there is a problem with CLT’s naivety in terms of the antecedent conditions that either facilitate or hinder this constructive process in theory. The theory assumes that an appropriately designed practice-based learning environment has been established, although it does not address how this can be achieved or under what conditions. In other words, CLT explains the process of learning in an ideal setting, but is less prepared to answer the question of why this type of learning environment was established.

Thirdly, TPB provides a necessary microfoundational approach by assuming that human behavior depends on intention, which is influenced by attitude, subjective norm, and perceptions of control [8,14]. In the education literature, TPB has been extensively used to describe students’ intentions to engage in entrepreneurship and learning behaviors [15]. However, TPB primarily explains behavioral intention and provides little insight into the learning process and its outcomes. It focuses on the student’s existing dispositions (self-efficacy, motivation, etc.) but takes no account of the learning environment in which these dispositions are stimulated, formed, or inhibited. It results in an excessively agentic, context-insensitive model that fails to consider how institutional and pedagogical conditions shape students’ chances of engagement and learning.

The three theoretical gaps, statism in RBV, contextual idealism in CLT, and agentic reductionism in TPB, together highlight the absence of an integrative theory that can account for the whole process from institutional resource allocation to the academic performance of students. It is exactly this theoretical gap that our research fills.

We suggest that these three apparently separate theories are, in fact, complementary rather than conflicting, and each provides an answer to a critical part of the causal chain in integrated education. The RBV provides the answer to “What are the resources?” (institutional input). The CLT provides the answer to “How does learning happen?” (cognitive construction). The TPB provides the answer to “Why do students engage?” (behavioral engagement). By combining all three theories, we create an innovative framework called “Resource Input-Cognitive Construction-Behavioral Engagement.”

In this regard, Instructional Practice Effectiveness becomes the key theoretical link that bridges RBV’s input resources with the learning processes in CLT. Specifically, we believe that institutional resources (teaching content, teacher’s competence, enterprise resources, and policy support) cannot lead to learning results directly but have to be properly combined into an effective practice, which would involve practical engagement, situational learning, and feedback [12,13]. Likewise, Student Engagement, guided by TPB, serves as the key behavioral driver of this teaching and learning process, shaping its outcomes and enhancing the effectiveness of instructional practices.

This integration yields a theoretical contribution that none of the individual theories can provide alone: a dynamic, multi-level account of how institutional inputs, pedagogical processes, and student agency co-produce integrated learning outcomes. This framework also enables us to empirically test a series of hypotheses about the direct and indirect pathways through which various factors are associated with Perceived Integration Outcomes, thereby moving beyond mere theoretical speculation to offer empirically grounded insights into the mechanisms of successful educational integration.

Based on this theoretical framework, the core constructs are defined as follows. Perceived integration outcomes refer to students’ subjective evaluations of improvements in innovative thinking, problem-solving ability, entrepreneurial intention, and interdisciplinary application in integrated courses [16,17]. Instructional practice effectiveness refers to the extent to which teaching processes facilitate the application of knowledge and the development of capabilities through practical engagement, situational learning, and interactive feedback [10,12]. Teaching Content refers to the structure of course knowledge and the design characteristics of learning tasks [16]. Teaching competence refers to teachers’ ability to integrate interdisciplinary knowledge and provide effective, practical guidance in integrated courses [18]. School-Enterprise Resources refer to external collaborative resources that support practice-based learning [5]. Policy Support refers to institutional arrangements that provide organizational and resource support for integrated education [19]. Student Engagement refers to students’ overall level of motivation, self-efficacy, and behavioral involvement in the learning process [15,30].

### 2.2 Research hypotheses

Within the “resource input-cognitive construction-behavioral engagement” framework, this study examines the relationships among key variables with a focus on instructional processes and their associations with perceived integration outcomes.

First, constructivist learning theory suggests that learning outcomes are closely related to learners’ participation in situated contexts and their engagement in knowledge construction [10]. In integrated education settings, instructional practice effectiveness facilitates knowledge integration and capability development through project-based and situational learning approaches, and is therefore expected to be positively associated with perceived integration outcomes.

- H1: Instructional practice effectiveness is positively associated with perceived integration outcomes.

Second, from the perspective of the resource-based view, teaching content, teaching competence, school-enterprise resources, and policy support are key resource inputs that are associated not only with learning outcomes but also with instructional processes [20]. Specifically, the integrative nature of teaching content supports the incorporation of practical activities [15–17]; teaching competence shapes instructional organization and facilitation [18]; school-enterprise resources provide authentic contexts and practical opportunities [5]; and policy support offers institutional and resource guarantees [19]. Accordingly, the following hypotheses are proposed:

- H2: Teaching content is positively associated with instructional practice effectiveness.
- H3: Teaching content is positively associated with perceived integration outcomes.
- H4: Teaching competence is positively associated with instructional practice effectiveness.
- H5: Teaching competence is positively associated with perceived integration outcomes.
- H6: School-enterprise resources are positively associated with instructional practice effectiveness.
- H7: School-enterprise resources are positively associated with perceived integration outcomes.
- H8: Policy support is positively associated with instructional practice effectiveness.
- H9: Policy support is positively associated with perceived integration outcomes.

Furthermore, following the “resource input-cognitive construction” logic, resource inputs are expected to be associated with learning outcomes through instructional processes. Therefore, instructional practice effectiveness may serve as a mediating mechanism linking resource inputs to perceived integration outcomes.

- H10: Instructional practice effectiveness mediates the relationship between teaching content and perceived integration outcomes.
- H11: Instructional practice effectiveness mediates the relationship between teaching competence and perceived integration outcomes.
- H12: Instructional practice effectiveness mediates the relationship between school-enterprise resources and perceived integration outcomes.
- H13: Instructional practice effectiveness mediates the relationship between policy support and perceived integration outcomes.

Finally, based on the theory of planned behavior, students’ motivation, self-efficacy, and willingness to participate are closely related to their level of engagement in learning activities and, in turn, are associated with learning outcomes [14]. Therefore, student engagement is expected to be associated with both instructional processes and perceived integration outcomes.

- H14: Student engagement is positively associated with instructional practice effectiveness.
- H15: Student engagement is positively associated with perceived integration outcomes.

In addition, Student engagement may be indirectly associated with perceived integration outcomes through instructional processes.

- H16: Instructional practice effectiveness mediates the relationship between student engagement and perceived integration outcomes.

### 2.3 Hypothesized model

**Fig 1:**
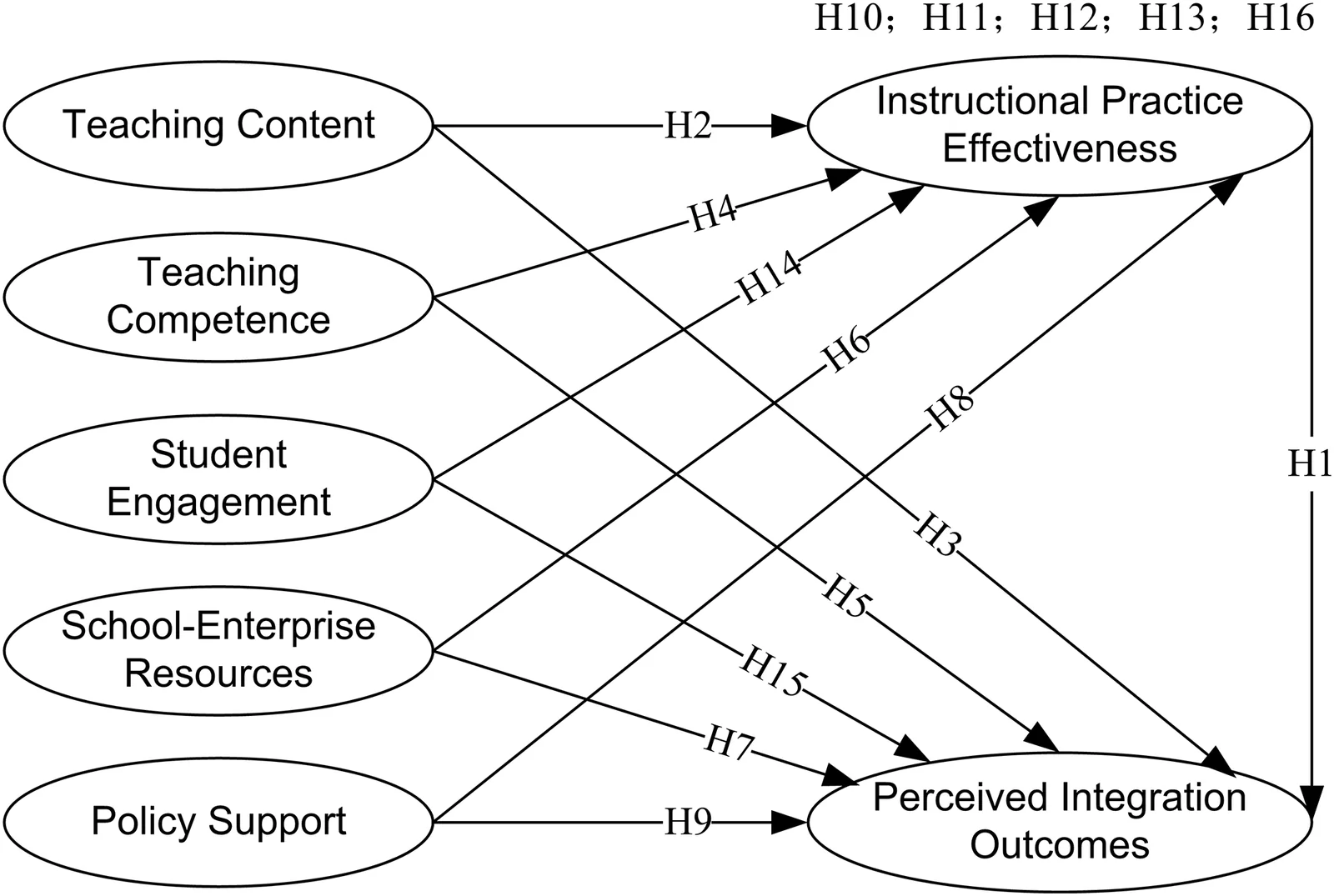
Hypothesized model of the pathways associated with perceived integration outcomes in the integration of professional education and IEE.

Based on the above analysis, this study proposes a hypothesized model (Fig 1) to illustrate the pathways associated with perceived integration outcomes in the integration of professional education and IEE. Teaching content, teaching competence, student engagement, school-enterprise resources, and policy support are expected to be associated with perceived integration outcomes both directly and indirectly through instructional practice effectiveness.

## 3 Data collection and methodology

### 3.1 Research design

This study adopted a quantitative, cross-sectional research design to examine the relationships among teaching content, teaching competence, student engagement, school-enterprise resources, policy support, instructional practice effectiveness, and perceived integration outcomes in the context of integrating professional education and IEE.

The research framework was developed by drawing on constructivist learning theory, resource-based theory, and the theory of planned behavior. Based on this framework, data were collected through a structured questionnaire using five-point Likert-scale measures adapted from established studies.

Structural Equation Modeling (SEM) was employed to test the proposed relationships because it enables the simultaneous assessment of measurement validity and structural associations among multiple latent constructs. The analytical procedure included reliability and validity assessment, confirmatory factor analysis (CFA), structural model estimation, and bootstrapping analysis for mediation testing.

Given the cross-sectional nature of the data, the results should be interpreted as associations among variables rather than definitive causal relationships.

### 3.2 Data collection

This study focuses on university students in Fujian Province, China, and employs a stratified random sampling approach to obtain the sample. Higher education institutions in Fujian were first classified into two categories: comprehensive universities (n = 39) and vocational colleges (n = 40). From each category, two institutions were randomly selected, yielding a total of 4 participating institutions.

With the support of these institutions, lists of enrolled students were obtained through student administration offices. A random sampling technique was employed, with 188 students selected from each university, for a total of 752 participants. The questionnaire was sent via official university email and learning management systems from 15 October to 15 December 2024.

To ensure high-quality data, respondents with more than 20% missing responses were excluded. Following data screening, 584 valid responses were retained, yielding a valid response rate of 77.9%.

### 3.3 Ethics statement

This research has been approved by the ethics committee of the institution where the authors work, before data collection, and is consistent with ethical guidelines. Before taking the survey, all participants received an informed consent statement that explained the study’s objective, the procedure, and how the data would be used. Participants’ electronic consent was obtained before completing the survey. While the majority of the sample comprises university students, there are some young participants (n = 23). Informed consent from their parents has been obtained through institutional channels. No personal details have been taken, and all data are anonymous.

### 3.4 Scale design

The survey questionnaire was developed by drawing on the mature scales of highly cited literature. The questionnaire was administered after the designing process to a sample of 75 undergraduate students through a small pilot test. Following this test, the researchers fine-tuned the questionnaire based on its outcome. In total, 40 questions were selected for the questionnaire. The questionnaire consists of two parts. The first part concerns basic demographic data, including respondents’ age, gender, and education level [21]. In the second section, the independent variables and mediating variables were included. The individual survey questions are listed in Table 1 below.

**Table 1:** Survey questionnaire.

Variable	Item	References
Basic Information	JB1: Gender: (1) Male (2) Female	Creswell, J. W., Creswell, J. D., 2017 [21]
	JB2: Age: (1) Under 18 (2) 18–22 (3) 23–27 (4) 28–32 (5) 33–40 (6) Over 40	
	JB3: Your major belongs to: (1) Science and Engineering, (2) Medicine, (3) Agriculture, (4) Humanities, (5) Other:	
	JB4: Your current level of study: (1) Associate Degree, (2) Undergraduate, (3) Master’s Degree, (4) Doctoral Degree, (5) Other:	
	JB5: The type of your university: (1) Comprehensive University, (2) Vocational and Technical College, (3) Other:	
Teaching Content	JX1: The curriculum at the university covers fundamental knowledge and core skills in entrepreneurship.	Neck, H. M., Greene, P. G., 2011 [22]
	JX2: The specialized courses integrate professional knowledge with real-world entrepreneurship cases.	
	JX3: The specialized courses include interdisciplinary content that effectively broadens our perspective on innovation.	
	JX4: The project tasks or assignments in the specialized courses are closely related to real-world entrepreneurial situations.	
	JX5: The content of the specialized courses aligns with current market and industry demands and is forward-thinking.	
Teaching Competence	JS1: Our instructors in specialized courses have extensive practical experience in entrepreneurship.	Rasmussen, E. A., & Sørheim, R., 2006 [23]
	JS2: Our instructors in specialized courses have an interdisciplinary background, enabling them to connect professional and entrepreneurial knowledge.	
	JS3: Our instructors in specialized courses place a strong emphasis on cultivating students’ innovative thinking throughout the teaching process.	
	JS4: Our instructors in specialized courses can provide effective entrepreneurial guidance and feedback.	
	JS5: Our instructors in specialized courses employ diverse teaching methods to intrigue learners’ curiosity.	
Student Engagement	XS1: I show a high level of interest and willingness to participate in entrepreneurship education courses.	Zhao, H., Seibert, S. E., & Hills, G. E., 2005 [15]
	XS2: I have a strong sense of self-efficacy and believe that I can complete entrepreneurial tasks.	
	XS3: I am willing to engage in collaborative learning and enjoy sharing innovative ideas with others.	
	XS4: I am able to demonstrate the application of interdisciplinary knowledge in the project activities of the course.	
	XS5: I have a high level of enthusiasm and curiosity for entrepreneurial activities.	
School-Enterprise Resources	QY1: The university enables us to participate in real entrepreneurial projects through collaboration with enterprises in our coursework.	Pittaway, L., & Cope, J., 2007 [24]
	QY2: The university provides us with abundant entrepreneurial resources, such as office space, technical equipment, and more.	
	QY3: Business mentors or external experts are involved in certain guidance components of the course instruction.	
	QY4: The university regularly organizes school-enterprise exchange activities to help us expand our entrepreneurial network.	
	QY5: The school-enterprise collaboration courses offer extensive internship and practical opportunities to enhance our entrepreneurial skills.	
Policy Support	ZC1: The university has specific policies in place to support the offering of entrepreneurship education courses.	Fayolle, A., Gailly, B., & Lassas‐Clerc, N., 2006 [25]
	ZC2: The university provides dedicated funding or entrepreneurship grants for entrepreneurial projects.	
	ZC3: The university has implemented policies to encourage our participation in entrepreneurial activities (e.g., credit transfer, entrepreneurial incentives, etc.).	
	ZC4: Our entrepreneurial projects can be recognized in academic assessments and may earn official grades or credits.	
	ZC5: The university has a comprehensive policy system that encourages us to participate in innovative and entrepreneurial endeavors.	
Instructional Practice Effectiveness	SJ1: The teaching process of specialized courses includes a significant number of practical components, such as project tasks, entrepreneurship competitions, and more.	Souitaris, V., Zerbinati, S., & Al-Laham, A., 2007 [13]
	SJ2: We are exposed to real entrepreneurial situations and problems during the learning process of specialized courses.	
	SJ3: In the teaching process of specialized courses, instructors encourage us to engage in independent thinking and propose innovative solutions.	
	SJ4: Throughout the course, there is frequent interaction between teachers and students, with instructors providing effective feedback on our entrepreneurial ideas.	
	SJ5: We have the opportunity to apply professional knowledge to solve real-world problems during the learning process and receive feedback from instructors.	
Perceived Integration Outcomes	RH1: The courses offered by the university effectively enhance our innovative thinking and problem-solving skills.	Martin, B. C., McNally, J. J., & Kay, M. J., 2013 [26]
	RH2: The entrepreneurship knowledge and skills we acquire in the courses have practical application value.	
	RH3: The courses offered by the university have increased our entrepreneurial intention, and we are more willing to pursue entrepreneurship in the future.	
	RH4: The courses offered by the university have enhanced our ability to apply interdisciplinary knowledge more effectively.	
	RH5: The courses offered by the university have helped us develop autonomous learning skills and increased our confidence in dealing with uncertain problems.	

### 3.5 Data analysis

Given that the variables examined in this study are latent constructs and that the relationships among them are relatively complex, structural equation modeling (SEM) was employed for data analysis and hypothesis testing [14,27]. SEM allows for the simultaneous estimation of measurement and structural models, making it suitable for examining relationships among multiple variables and latent constructs.

Specifically, a confirmatory factor analysis (CFA) was conducted first to assess the measurement model’s reliability and validity. Based on this, a structural model was developed to test the proposed hypotheses. Model parameters were estimated using the maximum likelihood method [28], and model fit was evaluated using commonly reported indices, including χ²/df, CFI, TLI, and RMSEA [28].

## 4 Results and discussions

### 4.1 Descriptive statistics

Five hundred eighty-four validated observations were obtained and presented in Table 2. The gender distribution of respondents and the distribution of students from comprehensive universities and vocational colleges were relatively even. Similarly, students in science and engineering, medicine, agriculture, and liberal arts (including management, literature, art, and other professional categories) were also relatively evenly distributed. 67.8% of participants were under 22, matching the profile of learners in higher education programs in China. Therefore, the collected sample data satisfies the study’s requirements.

**Table 2:** Descriptive statistics results.

Item	Category	Quantity	Percentage (%)
Gender	Male	264	45.2
	Female	320	54.8
Age	Under 18 years old	23	3.9
	18-22 years old	373	63.9
	23-27 years old	134	22.9
	28-32 years old	54	9.3
	33-40 years old	0	0
	Over 40 years old	0	0
Your current level of study	Science and Engineering	151	25.8
	Medicine	133	22.8
	Agriculture	147	25.2
	Humanities	153	26.2
Your current level of study	Associate Degree	204	34.9
	Undergraduate	192	32.9
	Master’s Degree	113	19.4
	Doctoral Degree	75	12.8
The type of your university	Comprehensive University	282	48.3
	Vocational and Technical College	302	51.7

### 4.2 Reliability test of the scale

This article used SPSS 24 to conduct a reliability analysis. The outcome demonstrates that the internal consistency reliability coefficient for the entire scale was 0.973, which meets the requirement of exceeding 0.7 [29]. All scale items exhibited corrected item-total correlation values exceeding 0.7, meeting the 0.5 or higher requirement [29]. The Cronbach’s Alpha coefficients for the observed variables after item deletion exceeded 0.9 and were lower than the overall scale’s Alpha value. Additionally, reliability analysis was conducted for each latent variable. Table 3 shows that the α values of all unobserved factors exceeded 0.9. The α coefficients of the observed variables after item deletion exceeded 0.8 and were lower than the α values of the corresponding unobserved variables. Therefore, the scale and observed indicators used in this study demonstrate high reliability.

**Table 3:** Reliability and validity analysis results.

Variable	Item	Cronbach’s Alpha if Item Deleted	Corrected Item-Total Correlation	Factor Loading	Cronbach’s Alpha	KMO	Significance	CR	AVE
Teaching Content	JX1	0.934	0.844	0.817	0.946	0.901	0.000	0.946	0.777
	JX2	0.933	0.854	0.833					
	JX3	0.936	0.832	0.804					
	JX4	0.934	0.847	0.814					
	JX5	0.928	0.880	0.861					
Teaching Competence	JS1	0.931	0.845	0.819	0.944	0.875	0.000	0.944	0.771
	JS2	0.935	0.819	0.780					
	JS3	0.931	0.844	0.818					
	JS4	0.932	0.836	0.805					
	JS5	0.923	0.889	0.869					
Student Engagement	XS1	0.921	0.852	0.824	0.938	0.909	0.000	0.938	0.753
	XS2	0.926	0.822	0.797					
	XS3	0.928	0.814	0.777					
	XS4	0.926	0.820	0.799					
	XS5	0.919	0.862	0.843					
School-Enterprise Resources	QY1	0.900	0.789	0.762	0.918	0.903	0.000	0.918	0.692
	QY2	0.900	0.790	0.764					
	QY3	0.902	0.776	0.738					
	QY4	0.900	0.787	0.763					
	QY5	0.897	0.802	0.773					
Policy Support	ZC1	0.941	0.835	0.803	0.949	0.863	0.000	0.948	0.784
	ZC2	0.934	0.873	0.857					
	ZC3	0.933	0.877	0.855					
	ZC4	0.943	0.822	0.789					
	ZC5	0.931	0.889	0.871					
Instructional Practice Effectiveness	SJ1	0.902	0.814	0.790	0.923	0.878	0.000	0.923	0.667
	SJ2	0.911	0.767	0.725					
	SJ3	0.910	0.774	0.730					
	SJ4	0.905	0.797	0.758					
	SJ5	0.897	0.841	0.822					
Perceived Integration Outcomes	RH1	0.966	0.934	0.920	0.974	0.923	0.000	0.974	0.882
	RH2	0.968	0.921	0.903					
	RH3	0.968	0.916	0.898					
	RH4	0.966	0.931	0.916					
	RH5	0.968	0.916	0.896					

### 4.3 Results of scale validity test

Factor analysis was performed on the collected data using SPSS 24. The KMO index for the entire scale was 0.966, with a significance value of 0.000. The KMO index for each variable exceeded 0.8, and each computed p-value was 0.000. These are considered acceptable because the KMO values were greater than 0.7 and the significance values were smaller than 0.05 [28]. Seven components were extracted using principal component analysis, aligning with the seven research variables presented in this article. The factor coefficients for each item were all greater than 0.7, meeting the requirement of exceeding 0.5 [28]. The specific values are presented in Table 3. After applying Kaiser’s normalized orthogonal rotation, the cumulative percentage of variance explained by the total factor loading was 81.690%, which meets the requirement of exceeding 80% [28]. Therefore, the seven extracted factors adequately represent the entire dataset.

The measurement model was constructed in AMOS 24, and confirmatory factor analysis was conducted. The $\frac{x^{\text{2}}}{df}$ was 2.411 (less than 3); NFI = 0.942, RFI = 0.936, IFI = 0.965, TLI = 0.962, and CFI = 0.965, which exceeds 0.9. The RMSEA value was 0.049, which is below 0.08. The model demonstrates a satisfactory fit when the value of $\frac{x^{\text{2}}}{df}$ is below 3 [27]. An RMSEA of less than 0.08 indicates good model fit [28]. Other indices, such as the Comparative Fit Index (CFI) and Tucker-Lewis Index (TLI), indicate better model fit as they approach 1. When these indices are above 0.9, the model fit is satisfactory [27]. Therefore, the data and model in this study exhibit a good fit.

In this study, key measures for convergent validity include Std. Estimate, Composite Reliability, and Average Variance Extracted [28]. In Table 3, the Std. Estimates for all items exceeded 0.8, the CRs exceeded 0.9, and the AVEs exceeded 0.6. Convergent validity is considered satisfactory when the CR exceeds 0.6, the AVE is above 0.5 for each variable, and the Std. Estimates for all items are greater than 0.5 [27]. Therefore, the data in this study demonstrate convergent validity and meet the required standards.

Discriminant validity is confirmed when a construct’s average variance extracted (AVE) demonstrates sufficient differentiation, as evidenced by its square root value exceeding the maximum correlation with other constructs in the model [30]. Discriminant validity assessment confirmed that all constructs’ AVEs consistently exceeded their cross-construct correlations, as shown in Table 4. Therefore, the seven variables examined, teaching content, teaching competence, student engagement, school-enterprise resources, policy support, instructional practice effectiveness, and perceived integration outcomes, have strong distinctiveness validity.

**Table 4:** Results of discriminant validity test for variables.

Variables	Perceived Integration Outcomes	Policy Support	School-Enterprise Resources	Student Engagement	Teaching Competence	Teaching Content	Instructional Practice Effectiveness
Perceived Integration Outcomes	**0.939**						
Policy Support	0.454	**0.885**					
School-Enterprise Resources	0.628	0.522	**0.832**				
Student Engagement	0.565	0.453	0.662	**0.868**			
Teaching Competence	0.63	0.529	0.783	0.697	**0.878**		
Teaching Content	0.586	0.463	0.656	0.583	0.683	**0.881**	
Instructional Practice Effectiveness	0.671	0.581	0.726	0.636	0.722	0.701	**0.817**

Given that this research relies on self-reported survey data from a single source, common method bias (CMB) cannot be overlooked. Therefore, multiple approaches were employed to assess this issue.

First, Harman’s single-factor test was conducted by performing an exploratory factor analysis of all the measurement items [31]. The results indicated that the first unrotated factor accounted for 28% of the total variance, which is below the commonly accepted threshold of 40%, suggesting that common method bias is unlikely to be a serious concern. Secondly, following the suggestions of Philip M. Podsakoff et al. (2003), a confirmatory factor analysis (CFA) was conducted to verify a single-factor solution in which all measurement items loaded on a single construct [32]. The results showed a poor model fit (e.g., for instructional practice effectiveness: χ²/df = 6.8, CFI = 0.62, TLI = 0.58, RMSEA = 0.12), which is substantially worse than that of the multi-factor model, further indicating that common method bias does not pose a serious threat to the validity of the findings.

Finally, various procedural measures were taken to address the methodological bias caused by questionnaire construction, such as ensuring respondent anonymity and randomizing the order of items to be measured, as recommended by Podsakoff et al. (2003) [32].

### 4.4 Results of structural equation modeling goodness-of-fit test

**Fig 2:**
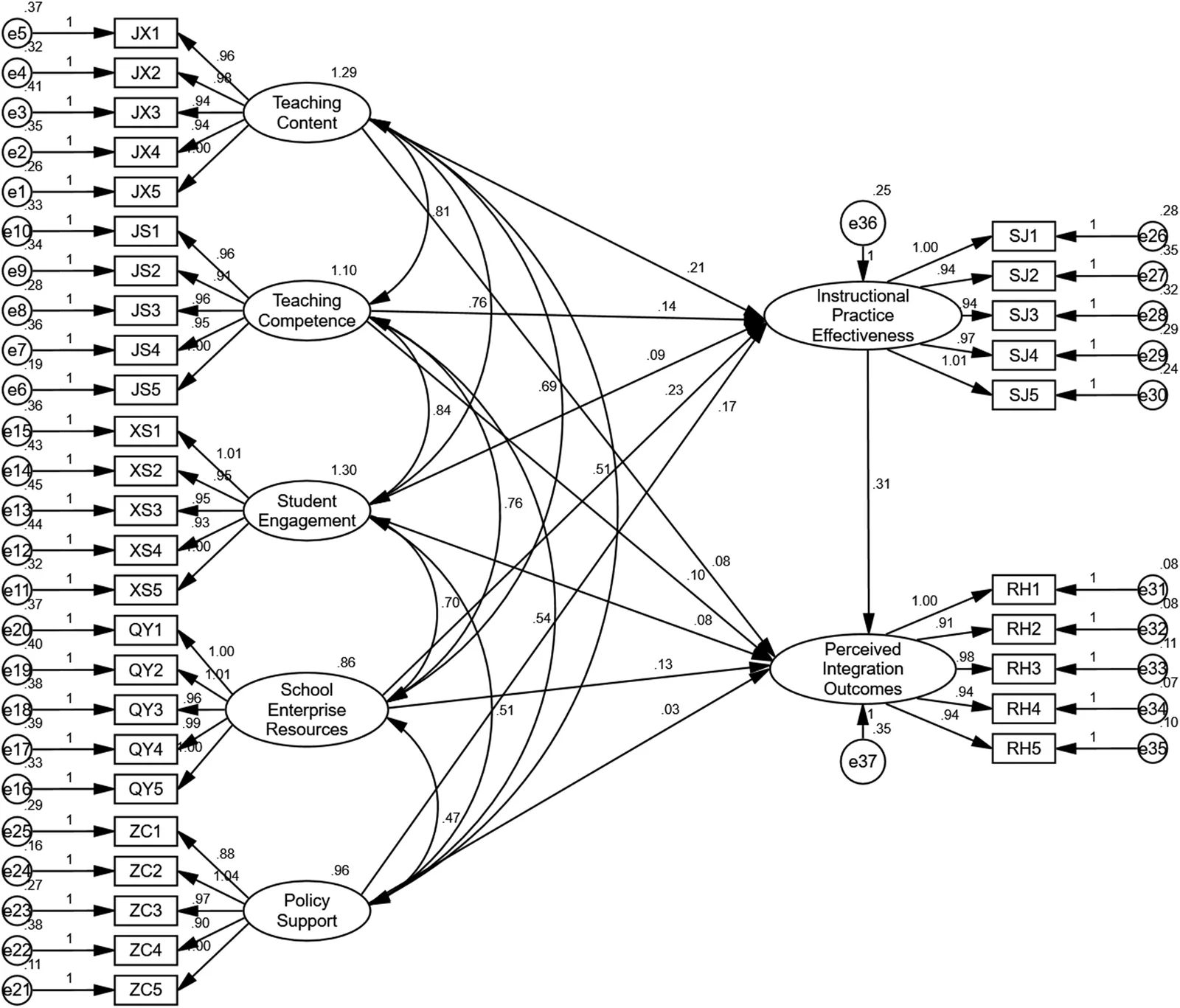
Structural equation model.

To analyze the pathway to perceived integration outcomes in the integration of professional education and IEE, this study adopted a Structural Equation Modeling (SEM) approach in AMOS 24. As shown in Fig 2, the measurement model demonstrated satisfactory fit indices ($\frac{x^{\text{2}}}{df}$=2.411, GFI = 0.885, AGFI = 0.866, IFI = 0.965, CFI = 0.965, TLI = 0.962, RMSEA = 0.049) [27].

### 4.5 Results of the direct effect test

The test of the direct effect hypothesis was conducted using Structural Equation Modeling in AMOS 26, and the results are presented in Table 5. Some of the indicators used in hypothesis testing are the critical ratio (C.R.), P-value, unstandardized coefficient, standardized path coefficient, and standard error. To qualify as a valid path, the critical ratio should be greater than 1.96, and the P-value should be less than 0.05 [33].

**Table 5:** Results of direct effect testing.

No.	Path	Estimate	Std Estimate	S.E.	C.R.	P	Results
H1	Instructional Practice Effectiveness→Perceived Integration Outcomes	0.307	0.314	0.058	5.264	0.000	Supported
H2	Teaching Content→Instructional Practice Effectiveness	0.211	0.276	0.032	6.510	0.000	Supported
H3	Teaching Content→Perceived Integration Outcomes	0.082	0.109	0.037	2.223	0.026	Supported
H4	Teaching Competence→Instructional Practice Effectiveness	0.135	0.164	0.046	2.933	0.003	Supported
H5	Teaching Competence→Perceived Integration Outcomes	0.102	0.126	0.050	2.023	0.043	Supported
H14	Student Engagement→Instructional Practice Effectiveness	0.088	0.116	0.033	2.705	0.007	Supported
H15	Student Engagement→Perceived Integration Outcomes	0.077	0.104	0.035	2.171	0.030	Supported
H6	School-Enterprise Resources→Instructional Practice Effectiveness	0.226	0.242	0.051	4.463	0.000	Supported
H7	School-Enterprise Resources→Perceived Integration Outcomes	0.131	0.144	0.056	2.339	0.019	Supported
H8	Policy Support→Instructional Practice Effectiveness	0.167	0.188	0.030	5.479	0.000	Supported
H9	Policy Support→Perceived Integration Outcomes	0.028	0.032	0.034	0.821	0.411	Not Supported

Overall, the findings provide support for the proposed direct associations. Teaching content, teaching competence, student engagement, school-enterprise resources, and policy support are significantly associated with instructional practice effectiveness. In addition, teaching content, teaching competence, student engagement, school-enterprise resources, and instructional practice effectiveness are significantly associated with perceived integration outcomes. However, the direct association between policy support and perceived integration outcomes is not significant.

### 4.6 Results of the mediation effect test

Mediation analysis employing bootstrapping procedures. The AMOS operation rule was set to random sampling, with a sample size of 5000, a 95% confidence interval, and the Bias-Corrected estimation method. Statistical significance of the mediation effect is determined when the confidence interval excludes zero, the SE value is less than 0.05, and the significance P-value is less than 0.05 [33]. The results of the mediation effect hypothesis testing in this study are shown in Table 6.

**Table 6:** Results of mediation effect testing.

No.	Path	Effect	SE	Bias-Corrected		P	Results
				95%CI			
H10	Teaching Content→Instructional Practice Effectiveness→Perceived Integration Outcomes	0.087	0.024	0.047	0.144	0.000	Supported
H11	Teaching Competence→Instructional Practice Effectiveness→Perceived Integration Outcomes	0.052	0.026	0.009	0.113	0.015	Supported
H16	Student Engagement→Instructional Practice Effectiveness→Perceived Integration Outcomes	0.036	0.016	0.012	0.074	0.006	Supported
H12	School-Enterprise Resources→Instructional Practice Effectiveness→Perceived Integration Outcomes	0.076	0.025	0.036	0.135	0.000	Supported
H13	Policy Support→Instructional Practice Effectiveness→Perceived Integration Outcomes	0.059	0.017	0.031	0.099	0.000	Supported

The findings indicate that instructional practice effectiveness significantly mediates the relationships between teaching content, teaching competence, student engagement, school-enterprise resources, policy support, and perceived integration outcomes.

### 4.7 Discussion

**Fig 3:**
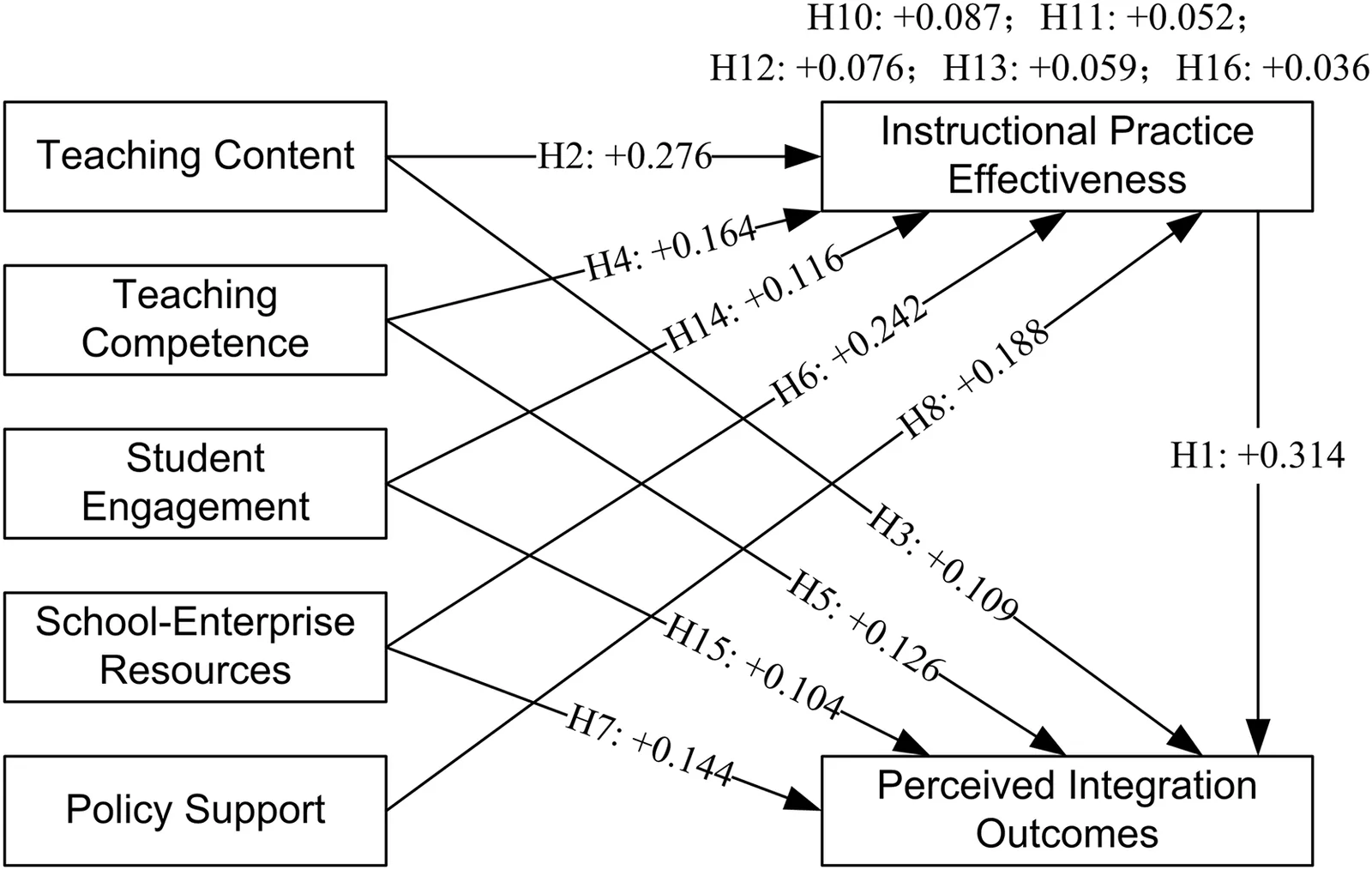
Formation pathway model of perceived integration outcomes in the integration of professional education and IEE.

Based on a sample size of 584 university students, this study investigates the pathway to the development of perceived integration outcomes in professional education and IEE integration. The results show that teaching content, teaching competence, student engagement, and school-enterprise resources positively affect perceived integration outcomes. The effects can be achieved through instructional practice effectiveness as a mediator. It demonstrates that the instructional process plays a critical role in transforming educational inputs into learning outcomes. The overall mechanism is illustrated in Fig 3.

(1) Why Does Instructional Practice Serve as the Central Transformative Mechanism?

The robust mediating role of Instructional Practice Effectiveness across multiple pathways (H10-H13, H16 supported) raises a fundamental question: why does the instructional process occupy such a pivotal position in integration education?

In other words, the answer to this question is found in the concept of entrepreneurial competence. Unlike declarative knowledge, entrepreneurship skills, opportunity identification, resource coordination, and risk evaluation are, by nature, procedural and context-dependent [13,25]. These competences emerge not through the passive transfer of information, but through active participation in the problem-solving process and reflection upon it [7,12]. This is exactly what is measured when instructional practices are effective. In other words, instruction practice is not only a tool for knowledge delivery but also a place where entrepreneurial cognition is constructed, a process that CLT theory has long explained but which has been scarcely examined in practice [10,11]. The findings also narrow down the usage of RBV within the education sector. Although RBV provides an understanding of why universities with greater resource capacity are more likely to succeed [6,20], our findings indicate that mere access to resources is not enough. Only through the proper orchestration of resources can student-centered pedagogies be generated, enabling effective resource utilization. It follows that instructional practice serves as a dynamic capability, turning static institutional capabilities into perceived value, which helps explain why similar institutions with identical capabilities produce very different results [2,4].

(2) Why Do External Supports Follow Different Pathways?

The different pathways for School-Enterprise Resources and Policy Support raise a perplexing question: Why do two types of support, which both seem to be positive, have relationships with outcomes through different mechanisms?

The impact of enterprise-school resources is directly related to their proximity. When enterprise mentors teach jointly, when students engage in industrial projects, or when an internship is an integral part of the course content, the external resource becomes instantly apparent and useful [5,24]. Students become aware of the link between theoretical knowledge and practical application; thus, relevance, one of the most important factors of learning outcomes, is increased [11]. These resources are valuable precisely because they are embedded in the instructional process rather than merely available at the institutional level.

On the other hand, the indirect effect of policy is related to the mechanism’s distant nature. Policies are applied at the macro-institutional level, providing the basis for a framework and incentives [34,35]. However, their path from this journey to the classroom passes through several intermediaries: administrative personnel who interpret the policy; department heads who allocate resources in accordance with it; and teachers who adapt their behavior to the new conditions. Each stage carries its own potential for conflict: misunderstanding, misaligned incentives, or faulty implementation [36]. This multi-layered mediation explains why students may be unaware of policy support even when it exists, and why such support may not directly shape their perceptions. The implication is not that policy is unimportant, but that its effectiveness hinges on implementation fidelity, a contingency often overlooked in policy design.

(3) Why Does Student Engagement Operate as Both Driver and Amplifier?

The dual ways in which Student Engagement influences outcomes, both directly (H15) and indirectly through instructional practices (H16), warrant further elaboration regarding this dual nature.

The direct relationship is consistent with one of the most basic tenets of TPB-namely, that those students who have high levels of entrepreneurial self-efficacy and positive attitudes will make greater efforts and be satisfied with their learning experiences [8,15]. This is a rather simple motivational process.

Theoretically, the indirect way is more interesting, as it suggests that engagement is not simply a personal trait but also a relational one. Highly engaged students tend to be more active participants in discussions, ask questions, and give constructive feedback to peers. This enhances instructional interactions for all students [13]. Seeing high levels of student interest, teachers are likely to respond with more challenging assignments and feedback tailored to individual needs, thereby increasing overall instructional efficiency. Such a mutual process means that engagement is a catalytic force that improves the transformational power of the instructional process. From a CLT perspective, engagement enables the social and collaborative aspects of knowledge construction, in which instruction shifts from instructor-centered to co-constructive [7,11].

(4) Theoretical Contributions

Synthesizing the above interpretations, three theoretical refinements emerge.

First, the consistent mediation through instructional practice offers a more precise specification of the “black box” between educational inputs and outcomes. Resources are effective to the extent that they are pedagogically activated. This refines the RBV by identifying pedagogical process as the dynamic capability that converts static assets into value, while also addressing a limitation of CLT, which often assumes supportive pedagogical environments without theorizing their institutional antecedents.

Second, the differential pathways of external supports highlight the importance of distinguishing proximal resources (embedded in instruction) from distal ones (operating at institutional levels). This distinction suggests that the effectiveness of external support should not be assumed based on its mere existence; rather, attention must be paid to the mechanisms of delivery and the extent of its connection to student experience.

Third, the dual role of student engagement extends TPB’s application from a purely individual-level model of behavioral prediction to a more contextualized account of how dispositions interact with instructional environments. This suggests that student characteristics and pedagogical practices co-evolve, and should be theorized as mutually reinforcing rather than independent.

## 5 Conclusion

### 5.1 Main findings

Based on the theoretical perspectives of constructivist learning theory, resource-based theory, and the theory of planned behavior, this study develops an integration pathway model of perceived integration outcomes through Structural Equation Modeling (SEM) using data collected from 584 university students, with integration of professional education and IEE considered. Based on the systematic perspective of “resource input, cognitive construction, behavioral engagement,” the relationship among important variables in the integration process can be clearly revealed in this study, and the following conclusions can be drawn:

(1) Several input variables contribute to the development of perceived integration outcomes, underscoring the multidimensional nature of integration education.
  The analysis indicates a significant relationship between teaching content, teaching competence, student engagement, and school-enterprise resources and the perceived integration outcome. It can be inferred from the above findings that the success of integration education is not solely based on content but also on teaching competence, student attributes, and the availability of external resources for practice.
(2) Instructional practice effectiveness is a fundamental mediation variable that connects theory to practice.
  The results also show that instructional practice effectiveness mediates the relationships among teaching content, teaching competence, student engagement, school-enterprise resources, and perceived integration outcomes. From these findings, it can be concluded that both input characteristics and process aspects of instructional practices determine integrated educational outcomes. Instructional practice effectiveness, as a process-related mediator variable, bridges knowledge building and competence formation, as well as resource coordination and behavior activation.
(3) Student Engagement involves behavioral and psychological preparedness, acting as both a precondition for cognition and as an anchor for behavior.
  The results show that students’ motivational factors, sense of self-efficacy, and intention to participate have direct relationships with integration outcome perception and indirect influences through instructional practice effectiveness. This supports the theory of planned behavior and confirms the important roles of behavioral intention and self-concept in influencing educational achievement. Consequently, integration education programs must focus more on building students’ motivation and intentionality.
(4) School-enterprise resources enhance contextual embeddedness and serve as critical external support.
  The study confirms that school-enterprise resources are significantly associated with perceived integration outcomes, particularly through their role in providing authentic learning contexts and facilitating resource co-creation within instructional practices. These findings extend the applicability of the resource-based view in educational settings, suggesting that enterprise resources should not merely serve as supplementary inputs but should be deeply integrated into curriculum design, practice implementation, and evaluation processes.
(5) The direct impact of policy support is negligible, as its influence is evident only through institutional and indirect mechanisms.
  Contrary to initial predictions, the direct correlation between policy support and perceived integration outcomes is statistically insignificant. This implies the presence of indirect policy influences and their delayed nature. This study suggests a gap between the formulation and implementation of classroom policies. The success of policy support does not lie within the scope of formal policy documents, but in how institutions convey these policies, teachers respond to incentives, and how resources are employed.

To conclude, the interaction between professional education and IEE should not be viewed merely as the sum of curricular knowledge or adopted policies. Rather, it represents a system of resource allocation, instruction, and student-led activities. The instructional process, being a nexus of resources, cognition, and behavior, provides the key channel for achieving perceived integration outcomes.

### 5.2 Practical implications

Based on the research findings and informed by the perspectives of constructivist learning theory, the resource-based view, and the theory of planned behavior, this study proposes the following practical recommendations for higher education institutions and policymakers to enhance the effectiveness of integration education:

(1) The curriculum system should focus on real-world situations and skill development, shifting from knowledge imparting to ability generation.
  Based on constructivist learning theory, the design of course content should strengthen the teaching concepts of “contextualization”, “task-oriented”, and “interdisciplinary integration”. Colleges and universities should systematically integrate innovation and entrepreneurship modules into professional courses. Students are guided to combine professional knowledge with market demands and technical application scenarios to build a knowledge system that solves real-world problems. Especially during the course development stage, it is necessary to thoroughly investigate students’ entrepreneurial interests and industry trends to ensure dynamic updates and a demand-oriented approach to the course content.
(2) Strengthen the development of teachers’ dual capabilities and promote the construction of a teaching community integrating “major + entrepreneurship”.
  Teachers are not only disseminators of knowledge, but also designers of the learning process and guides of practice. Colleges and universities should enhance teachers’ design, guidance, and evaluation abilities in innovation and entrepreneurship education through specialized training, enterprise practice, and other forms. It is suggested to establish a collaborative teaching mechanism of “on-campus mentors + enterprise mentors”, cultivate a teaching team with cross-border thinking and practical experience, and achieve the organic integration of educational resources and industrial resources.
(3) Student-centered, stimulate their willingness to learn and self-efficacy, and enhance the behavioral foundation for educational integration.
  Based on the theory of planned behavior, students’ learning motivation, self-identity, and entrepreneurial intention are significantly positively associated with Perceived Integration Outcomes. Colleges and universities should enhance students’ active participation through diverse means such as personalized learning support, project-based learning, group collaboration, and outcome presentation. Thus, improve students’ emotional connection to and behavioral drive for the course content. Meanwhile, by setting up incentive mechanisms such as innovation credits and entrepreneurship points, students can be guided to develop a continuous, committed learning behavior in practice.
(4) Build a sustainable school-enterprise collaborative education mechanism to achieve resource co-construction and value co-creation.
  According to the resource-based theory, enterprise resources are key external variables that support educational integration. Colleges and universities should take the initiative to establish strategic cooperative relations with local industries and innovative enterprises. Thus, cooperation carriers such as production-education integration platforms, training bases, and incubation projects will be promoted to enhance students’ learning experience in a real business environment. It is suggested that universities guide enterprises to more deeply participate in course development, project guidance, and outcome evaluation through institutional design, thereby realizing the functional embedding of enterprise resources in the teaching process.
(5) Optimize the policy supply system and promote the transformation of educational governance from policy formulation to implementation guarantee.
  Research shows that the perceived effectiveness of policy support is low, suggesting deficiencies in its implementation process. Policy makers should enhance the pertinence of policy promotion and the rigidity of system implementation. Specifically, the means of communication, participation requirements, and implementation procedures for both teachers and students should be further improved. Universities can establish a policy service specialist or an entrepreneurship guidance center to facilitate connections between policy resources and teaching activities. This would improve the efficiency of policy implementation and its effectiveness for students.

The implementation of a combination of specialized and innovative education should not only be achieved through course and resource integration, but also through the transformation of educational ideas, methods, and coordination. From the practical implications of this study, it can be concluded that deep integration of specialized education and IEE can be achieved only by working together across the five aspects of curriculum, teacher, student, resource, and policy.

### 5.3 Limitations and future research

Despite this study’s contributions through theoretical and empirical approaches, several limitations should be considered for further research. First, because this paper uses cross-sectional data, the ability to establish causal relationships between variables might be limited. For instance, future research can employ either longitudinal data or multiple data sources to make its findings even more robust and realistic. Second, given the sampling approach, the research is biased toward data from students across various educational settings. Thus, future research can address this limitation by employing a sample covering broader geographical and institutional settings to increase external validity. Third, regarding measurement, perceived integration outcomes in this study are determined through learners’ subjective perceptions. Even though it enables capturing the true learning experience, such a measure is different from performance-based assessments. To further address this issue, future studies might consider objective outcome measures and multiple assessment approaches.
